# FECC-Net: A Novel Feature Enhancement and Context Capture Network Based on Brain MRI Images for Lesion Segmentation

**DOI:** 10.3390/brainsci12060765

**Published:** 2022-06-11

**Authors:** Zhaohong Huang, Xiangchen Zhang, Yehua Song, Guorong Cai

**Affiliations:** 1 Computer Engineering College, Jimei University, Xiamen 361021, China; zhaohong@jmu.edu.cn (Z.H.); xiangchenzhang@jmu.edu.cn (X.Z.); 2 The Second Affiliated Hospital of Xiamen Medical College, Xiamen 361021, China

**Keywords:** medical image segmentation, brain MRI image, deep learning

## Abstract

In recent years, the increasing incidence of morbidity of brain stroke has made fast and accurate segmentation of lesion areas from brain MRI images important. With the development of deep learning, segmentation methods based on the computer have become a solution to assist clinicians in early diagnosis and treatment planning. Nevertheless, the variety of lesion sizes in brain MRI images and the roughness of the boundary of the lesion pose challenges to the accuracy of the segmentation algorithm. Current mainstream medical segmentation models are not able to solve these challenges due to their insufficient use of image features and context information. This paper proposes a novel feature enhancement and context capture network (FECC-Net), which is mainly composed of an atrous spatial pyramid pooling (ASPP) module and an enhanced encoder. In particular, the ASPP model uses parallel convolution operations with different sampling rates to enrich multi-scale features and fully capture image context information in order to process lesions of different sizes. The enhanced encoder obtains deep semantic features and shallow boundary features in the feature extraction process to achieve image feature enhancement, which is helpful for restoration of the lesion boundaries. We divide the pathological image into three levels according to the number of pixels in the real mask area and evaluate FECC-Net on an open dataset called Anatomical Tracings of Lesions After Stroke (ATLAS). The experimental results show that our FECC-Net outperforms mainstream methods, such as DoubleU-Net and TransUNet. Especially in small target tasks, FECC-Net is 4.09% ahead of DoubleU-Net on the main indicator DSC. Therefore, FECC-Net is encouraging and can be relied upon for brain MRI image applications.

## 1. Introduction

Chronic stroke is an acute cerebrovascular disease with a high disability rate and high mortality rate. At present, chronic stroke is one of the most common diseases, and the number of deaths due to chronic stroke each year accounts for a large proportion [1,2]. Active prevention and treatment can minimize the mortality rate; thus, clinical intervention is necessary for the treatment of chronic stroke patients. Clinical treatment of chronic stroke requires the use of magnetic resonance imaging (MRI) to present the brain structure to assess brain behavior and formulate corresponding treatment plans. However, assessing the patient’s condition requires accurate positioning of the lesion area, which is a time-consuming and labor-intensive process. Therefore, automatically completing the identification of the diseased area by computer is used to assist the doctors in more accurately judging the patient’s condition.

With the rapid development of deep learning, convolutional neural networks (CNNs) have played an indispensable role in the field of medical segmentation. In particular, the encoder–decoder architecture represented by U-Net is widely used [3,4,5,6]. However, the traditional encoder–decoder framework cannot flexibly balance the precise localization of lesions and the sufficient extraction of contextual information, which will lead to an inaccurate detection of lesions of different sizes and blurred recovery of lesion boundaries. On the practical application level, the small-parameter medical segmentation model can effectively speed up the network inference process, realize rapid localization of lesions, and reduce the time cost of clinical treatment. However, current medical image segmentation models cannot have the characteristics of small parameter scale and excellent segmentation performance at the same time.

Combining the two aforementioned reasons, this paper proposes a medical image segmentation model with small parameters and excellent performance that conforms to the application scenario. This paper has made the following contributions:

(1) In view of the problem that the current medical image segmentation model parameters are large and cause the inference speed to be limited, we propose a new encoder–decoder structure. This work greatly reduces the model parameters and ensures that both shallow surface features and deep semantic features are fully extracted.
(2) The current mainstream medical image segmentation encoder inevitably produces loss of the shallow edge features in the process of downsampling. Therefore, we propose an enhanced encoder composed of depthwise separable convolutions to enhance the boundary features and effectively restore the lesion boundary.
(3) Medical images usually present imbalanced foreground information and background information, which causes the network training to fall into a local optimal state. This paper proposes hybrid loss to effectively solve the aforementioned problems and accelerate the model’s convergence speed.
(4) The proposed model’s effectiveness was evaluated on an open-source dataset, ATLAS, based on MRI images, and the results show that our network is superior to state-of-the-art methods and can be used as a baseline for brain MRI image segmentation.

## 2. Related Work

Due to the powerful calculations of computers, image segmentation algorithms based on deep learning have become the most essential tool for automatically locating lesion information. In 2015, Long J et al. [7] proposed a fully convolutional network (FCN), which proved that an end-to-end network can be implemented for segmentation problems. Therefore, the FCN is called the pioneer of segmentation algorithms. The U-Net architecture was designed by Ronneberger et al [8]. The U-Net architecture effectively improves the segmentation performance of medical datasets and proposes an effective method for processing large-size images. Chen et al. [9,10] proposed the DeepLab to improve the segmentation network without increasing the amount of parameters. In particular, DeepLab V3 [11] improves the ASPP in the spatial dimension and improves the performance of the previous version. Lin et al. [12] proposed that RefineNet optimizes the large number of calculations generated by dilated convolution and designs all components following the residual connection method. Jha et al. [13] stacked two U-Net architectures to form a novel architecture called DoubleU-Net. This method was evaluated on four different medical datasets, showing its superiority in semantic information extraction. Chen et al. [14] proposed TransU-Net that combines a transformer [15,16,17] and U-Net, adopting the transformer’s global adaptation to solve the limitations of convolution operations for long-range modeling. Additionally, the proposed method achieved excellent results in multi-organ segmentation.

Chronic stroke is a disease in which brain tissue is damaged due to the sudden rupture of blood vessels in the brain or the obstruction of blood vessels leading to the inability of blood to flow into the brain. A severe stroke will cause permanent nerve damage. If not diagnosed and treated in time, it will cause serious complications and even death. At present, large-scale neuroimaging studies are optimistic about the prospects of stroke recovery and point out the potential of deep learning. As we know, deep learning algorithms require large datasets to optimize performance. Therefore, a large number of medical datasets [18,19] are open sourced. This paper presents ATLAS [20] based on brain MRI images with manually segmented lesions and metadata.

From the aforementioned works, we can observe that there have been substantial efforts to link segmentation algorithms with medical imaging. In recent years, these works have been widely applied to the processing of brain MRI images. In 2021, Kadry et al. [21] designed a modified moth–flame optimization algorithm based on Kapur‘s thresholding and a chosen segmentation technique for further achievement in tumor extraction. The algorithm is suitable for the segmentation of high-density brain tumor lesions. Still, when the difference between the pixel grayscale of the foreground and the image background is slight, the performance of the threshold algorithm will be affected. For example, the segmentation of low-density brain infarction lesions will have the problem of missing localization. Kadry et al. [22] solved the problem of localization of brain lesions through a deep learning algorithm and proved the feasibility of U-Net in brain MRI image segmentation. Later, Maqsood et al. [23] proposed edge detection based on fuzzy logic regarding brain MRI images, and then input the pre-processed images into the U-Net network for detection. By comparing the latest brain MRI lesion segmentation methods, some advances in the current work can be summarized: (1) Traditional machine learning methods are suitable for the segmentation of high-density lesions (brain tumors) but not for low-density lesions (cerebral infarction). (2) The current deep learning-related algorithms focus on the pre-processing and post-processing of brain MRI images to optimize the indicators of U-Net, but similar optimization strategies are cumbersome and still retain the shortcomings of U-Net. This paper proposes a novel medical image segmentation model that can solve the problem of insufficient U-Net context information, feature information extraction, and skip the tedious image preprocessing process. It is worth mentioning that FECC-Net can show better segmentation performance in brain MRI images.

## 3. Method

**Figure 1:**
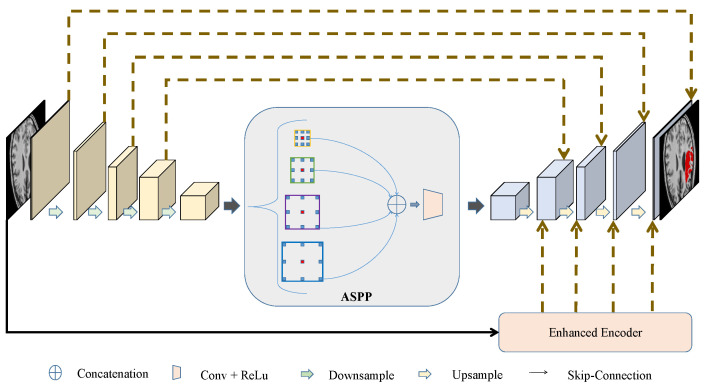
The overall structure of the proposed FECC-Net. FECC-Net is mainly composed of an encoder–decoder structure, an ASPP model, and an enhanced encoder.

The proposed FECC-Net architecture is illustrated in Figure 1. FECC-Net is mainly composed of an encoder–decoder structure and a skip connection. Considering that high-quality multi-scale features are the key to improving segmentation performance, we add an ASPP module at the interface of the encoder and decoder. The ASPP model integrates the features of different receptive fields through multiple convolutions of diverse scales to capture more refined context features. In addition, we design an enhanced encoder composed of depthwise separable convolutions, which is used for secondary skip connection operations to ensure that the deep semantic information of the network is extracted and to enhance boundary features, which is beneficial to the detection of small-scale objects and recovery of lesion boundaries.

### 3.1. Encoder–Decoder Architecture

There have been many experiments indicating that deeper neural networks bring adequate performance for medical image segmentation, but a network with too many layers may encounter problems, such as huge parameters and gradient explosions, which are not suitable for practical applications. We propose a new encoder–decoder architecture that solves the aforementioned problems and obtains better results.

This paper designs an ultra-light encoder in which each encoder block only needs to perform two 3 × 3 convolution operations, each followed by a batch normalization. The batch normalization reduces the internal co-variant shift and also regularizes the model. A rectified linear unit (ReLU) [24,25,26,27] activation function is applied. Two advantages accompany the ReLU activation function: (1) It realizes the sparse representation of the network. The ReLU activation function outputs the true zero value of the negative input, which effectively prevents the training from overfitting and speeds up the network convergence speed. (2) The value range of the ReLU activation function is not easily saturated, which can completely avoid disappearance of the gradient during training. In medical image segmentation, the sparsity of the ReLU activation function effectively reduces the error caused by overfitting, and the Relu activation function keeps the gradient proportional to the node activation, continuously optimizing the model and improving segmentation accuracy. This is followed by a squeeze-and-excitation block [28], which suppresses the redundant extraction of features and enhances the quality of the feature maps.

U-Net is the benchmark for medical image segmentation, but its ability to obtain image context information is limited. In this paper, the output of the high-resolution features by the encoder is input into atrus spatial pyramid pooling (ASPP). ASPP can integrate semantic information of different scales and fully capture context information. Finally, the depth features fused with multi-scale information are upsampled to restore the spatial features. The experimental results show that the network’s main structure proposed in this paper greatly reduces the amount of calculation and significantly improves the performance.

### 3.2. Enhanced Encoder

**Figure 2:**
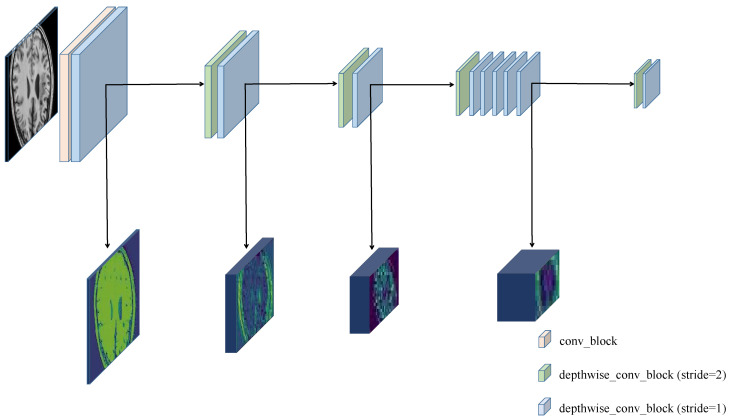
Architecture of the proposed enhanced encoder for feature enhancement.

The encoder–decoder architecture combines the intermediate features of the encoder with the decoded output via skip connections, allowing the network to obtain high-level semantics and low-level fine-grained surface features. We inherited this contextual information fusion strategy. However, simple feature connections cannot fully recover the loss of object boundary information during downsampling. Therefore we propose a novel encoder for boundary feature enhancement. Figure 2 is the overall architecture of the enhanced encoder. The main components of the encoder are the convolution block and depthwise convolution block. The convolution block adopts a 3 × 3 convolution, followed by batch normalization and the ReLu activation function. The depthwise convolution block uses a 3 × 3 depthwise separable convolution and a 1 × 1 convolution operation, and both operations are followed by batch normalization and the ReLu activation function. Compared with the shallow encoder, the enhanced encoder adopts successive separable convolutions for a stacking combination, which increases the depth of the network, preserves the obvious features that exist in the shallow layers, and helps to better preserve image properties. In addition, separable convolutions are more sensitive to shallow features and do not interfere with the deep features extracted by the main encoder.

### 3.3. Hybrid Loss Function

In MRI images of the brain, the lesion area of stroke disease is usually small. In the training process, much background information may dominate the direction of model training and eventually fall into a local optimal state. Therefore, a suitable loss function is particularly important. For the ATLAS dataset, we propose a hybrid loss function to balance the influence of the foreground factor and the background factor on the training trend.

#### 3.3.1. Binary Cross Entropy Loss

The binary cross entropy (BCE) loss is often used as a binary classification problem, which can measure the similarity between the real result and the predicted result. In particular, the use of the sigmoid function in the gradient descent can avoid the problem of a reduction in the learning rate of the mean square error loss function, so that the learning rate can be adjusted reasonably according to the output. In the brain MRI images, BCE loss makes the model fit correctly and prevents the model from entering the local optimal state in the initial training stage. As shown in Equation (1), *y* represents the distribution of real marks, and $\hat{y}$ is the predicted mark distribution of the trained model. *N* represents the number of images in the training set during the training process and represents the number of images in the validation set during the validation set process, and *i* represents the number of images in the corresponding dataset.

(1) $$L_{BCE}\left(y,\hat{y}\right)=-\sum_{i=1}^{N}y_{i}\ln{\hat{y}}_{i}+\left(1-y_{i}\right)\ln\left(1-{\hat{y}}_{i}\right)$$

#### 3.3.2. Dice Coefficient Loss

In the brain MRI images, the number of healthy samples is greater than that of stroke samples. The unevenness of positive and negative samples causes the model to be more sensitive to irrelevant information while ignoring the extraction of main features. Therefore, we introduced dice loss to optimize the sample imbalance. As shown in Equation (2), *y* represents the ground truth, and $\hat{y}$ is the predicted mask of FECC-Net. Among them, *N* represents the number of images in the training set or validation set. δ is an adjustable parameter, which is used to prevent the error of division by zero and to cause negative samples to have a gradient propagation. Generally, we set the adjustable parameter to a value close to 0 (from 1 × 10${}^{-15}$ to 1 × 10${}^{-13}$), which will not affect the convergence of the network but also ensures that information from the negative samples can be harvested in backpropagation to adjust the network weight for better recall.

(2) $$L_{\mathrm{Dice}\;}\left(y,\hat{y}\right)=1-\frac{2\sum_{t=1}^{N}y_{i}{\hat{y}}_{i}+\delta }{\sum_{i=1}^{N}y_{i}^{2}+\sum_{i=1}^{N}{\hat{y}}_{t}^{2}+\delta }$$

#### 3.3.3. Proposed Hybrid Loss

We propose the hybrid loss (HL) function based on the two loss functions above and use their L2 paradigm as a new loss function to optimize the gradient change of each iteration and speed up the convergence time of training. The formula for the hybrid loss function is shown in Equation (3).

(3) $$HL\left(y,\hat{y}\right)=\sqrt{L_{BCE}{\left(y,\hat{y}\right)}^{2}+L_{Dice}{\left(y,\hat{y}\right)}^{2}}$$

## 4. Experiments

### 4.1. Evaluation Metrics

The most common evaluation methods in the field of deep learning are recall and precision. Precision evaluates the quality of the segmentation, as the proportion of boundary pixels in the predicted result that correspond to boundary pixels in the ground truth of the image. Recall usually reflects the extent of recall in the lesion area, which can evaluate the ability of the model to detect lesions. To show the performance of FECC-Net more clearly, we decided to add DSC as the main indicator, which can measure the similarity between two sets. In medical image segmentation, DSC measures the overlap between predicted and annotated pixels. In addition, we introduce the mIoU indicator for auxiliary evaluation, which can effectively reflect the effect of edge segmentation. The current medical segmentation model pursues fewer parameters, so we emphasize calculation of the total parameters. In all tables, we indicate the computational metrics of FECC-Net with bold values.

### 4.2. Dataset

The Anatomical Tracings of Lesions After Stroke (ATLAS) dataset [20] is a challenging 3D medical image dataset. To verify the excellent performance of our method, we adopted it as the dataset. A total of 229 patients with chronic stroke were scanned with the MRI T1 sequence in the ATLAS dataset. The size of each case was 233 × 197 × 189. Sequence scans were extracted with the help of the ITK Snap tool and used basic axial 2D slices [29] for a total of 43,281 slices. We divide the dataset into a training set, a validation set, and a test set in a ratio of 8:1:1. The training sample has 33,264 slices, which can ensure that the model learns enough information. The test sample has 4158 slices. In order to verify the effectiveness of FECC-Net under various segmentation tasks, we grouped the test sample according to the number of pixels of the real mask in the dataset and divided them into small-sized lesion tasks of 0–100 pixels (excluding the lesion-free slices), 100–1000 pixels for medium-sized lesions tasks, and 1000-pixel-plus for large-sized lesions tasks. Among them, there were 142 slices of small-sized lesions, 361 slices of medium-sized lesions, and 418 slices of large-sized lesions.

### 4.3. Experiment Setup

In our experiments, we used the Keras framework [30] with Tensorflow 2.5.0 [31] as the backend. Without affecting the spatial information of the original image, we uniformly adjusted the size of the image to 512 × 512 to balance the training time and complexity. In the selection of the model optimizers, FECC-Net adopted the Adam optimizer to replace the traditional SGD optimizer. The most important feature of Adam is that it can adapt the learning rate and prevent the model from falling into a local optimal solution in the process of gradient descent. Therefore, we set a large learning rate of $1\times e^{-4}$ before training to make the model have more momentum in the initial stage. After the model has become gradually stable in the later stage, the adaptive learning rate gradually decreases with the number of iterations. To speed up the convergence of the model, the batch size of the network was set to 16. The default model accepts 300 iterations, but different computing resources have different requirements for the number of iterations. The model can be set to end the training at the time of automatic convergence. It is worth mentioning that we randomly sampled the training samples and performed random data enhancement operations (elastic transformation, rotation transformation, noise addition, etc.) on the sampled images. When the number and form of training samples are rich, the model has a strong generalization ability and can effectively compensate for the overfitting problem.

## 5. Results

In this section, we will show the segmentation process of FECC-Net and compare our proposed model with the mainstream medical segmentation model. We also evaluated three different tasks for the ATLAS dataset and show the qualitative results to prove the superiority of FECC-Net. To verify the influence of different components of FECC-Net on segmentation performance, we conducted ablation experiments on enhanced-encoder and loss function. Considering the real-life application of the model, this paper conducts noise addition experiments on images, discusses the robustness of our proposed method to noise.

Our quantitative results on the ATLAS dataset are summarized in Table 1. The experimental results show that the proposed FECC-Net achieved the best performance, considering the value DSC, Recall, and Precision. It is worth noting that our model parameters are smaller than other models, which speeds up the convergence of the model. Regarding the DSC score, FECC-Net ranks first with a score of 0.6789, which is 8.97% higher than DoubleU-Net.

### 5.1. The Performance on Different Tasks

In Table 2, we quantitatively compare our model to four currently advanced methods in the small target task. FECC-Net has the highest scores on the main indicators (DSC and mIoU). The DSC and mIoU of FECC-Net are 4.09% and 4.45% higher than that of DoubleU-Net, respectively. This shows that our proposed method could achieve a promising segmentation performance in the small target task.

**Figure 3:**
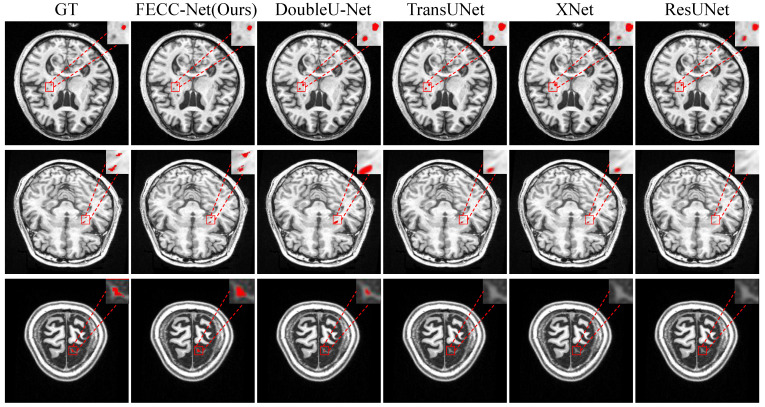
Qualitative result on small target from ATLAS. Typical small targets of stroke are difficult to find with the naked eye.

As shown in Figure 3, small target lesions in brain MRI images are difficult to distinguish with the naked eye and are mainly divided into two cases: missed detection and false detection. The first case in Figure 3 shows that other medical image segmentation models are prone to mislocalization when encountering dark pixels, and FECC-Net shows good performance. Hybrid loss plays an important role; it is effective. The training of the avoidance model tends to interfere with regions. The last two cases in Figure 3 mainly have the problem of missed detection. There are two reasons for such a problem: (1) insufficient extraction of deep features or (2) insufficient extraction of contextual information. FECC-Net does not experience loss of small target localization because the enhanced encoder ensures the depth of the network and its ASPP structure effectively capture contextual information through multi-scale fusion.

**Figure 4:**
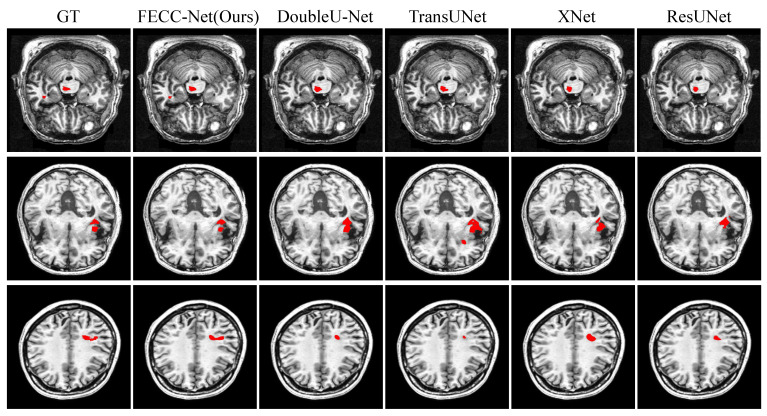
Qualitative result on medium target from ATLAS. Typical medium targets of stroke are often detected abnormally in discrete lesions and elongated lesions.

The second case was conducted on the medium target from ATLAS. From Table 3, we can see that FECC-Net outperforms TransUNet by 2.96% in DSC and 4.02% in mIoU on the medium target from ATLAS. As can be observed from Figure 4, the medium targets are mainly divided into two cases: the first case is the scattered lesions, and the second case is the slender lesions. In scattered lesions, the phenomenon of missed detection occurs easily when the dispersion distance is long, and it is easy to predict the scattered lesions as a connected overall lesion when the dispersion distance is short. In slender lesions, loss of lesion prediction is common. It turns out that both TransUNet and DoubleU-Net have the aforementioned series of problems. However, FECC-Net extracts context information while ensuring depth information, so it is more accurate for the localization of lesions.

**Figure 5:**
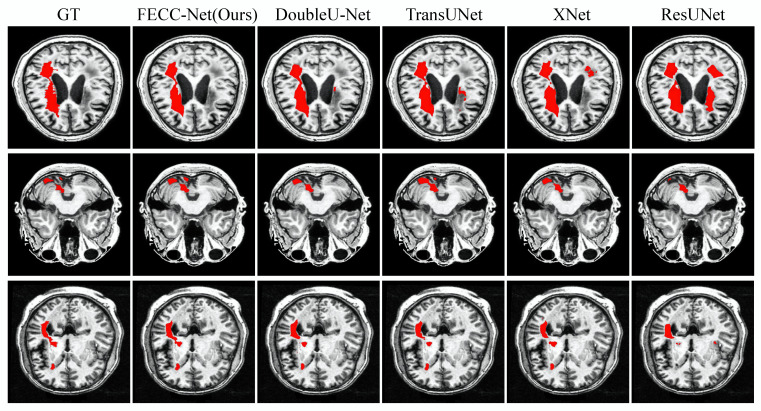
Qualitative result on large target from ATLAS. Typical large-target tasks in stroke are easily disturbed by shadowed areas, resulting in abnormal recovery at the edge of the lesion.

The third experiment was conducted on the large target task. As indicated in Table 4, FECC-Net achieves a DSC of 0.9072 and mIoU of 0.8311, which are 1.85% and 1.39% higher than those of DoubleU-Net, respectively. The large object task is the most common case in life, which fully reflects the advantages of our proposed method. The three cases in Figure 5 show three challenges that are prone to large-objective tasks: (1) interference from redundant information, (2) loss of localization of discrete lesions, and (3) abnormal recovery of the edge of the lesion. The proposed algorithm solves the problem of low recall caused by redundant information through hybrid loss, solves the problem of discrete lesion localization via feature fusion of different scales through the ASPP structure, and solves the problem of abnormal boundary restoration through the enhanced encoder to enhance boundary features.

### 5.2. Enhanced Encoder Validity

**Figure 6:**
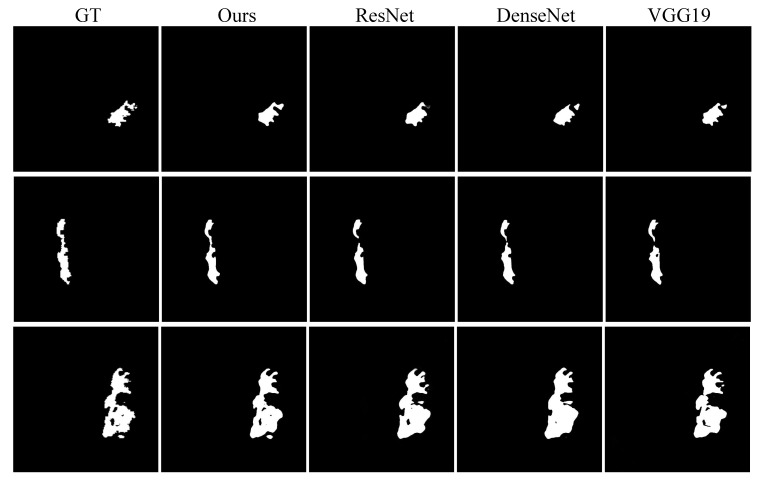
Qualitative result of four enhanced encoders.

The motivation of the enhanced encoder is to compensate for the loss of apparent information caused by the downsampling process of the image and to achieve the enhancement of boundary features. To verify the effect of different baseline feature enhancements, we evaluated VGG19 [38], ResNet [39], and DenseNet [40] as the enhanced encoder of FECC-Net, respectively. From the quantitative analysis of the four enhanced encoders in Table 5, we can observe that different additional encoders all achieve sufficient performance, which proves that the enhancement of boundary features is beneficial to the recovery of lesion information. Two major challenges for medical image segmentation models in the process of boundary recovery include: (1) The boundary of the lesion is tortuous, resulting in abnormal recovery, and the lesion is divided into two parts (case 1 and case 2 in Figure 6). (2) The discrete lesions are close to the large target lesion, and ignoring the discrete edge details leads to a lack of recovery of the lesions (case 3 in Figure 6). The enhanced encoder of FECC-Net shows excellent performance under both challenges; the main reason is the depthwise separable convolution is more sensitive to the extraction of apparent features and does not destroy the deep features extracted by the main encoder. Boundary information is extremely important in the clinical diagnosis of cerebral infarction, and doctors can predict the spread of cerebral infarction through the edge of the lesion. Our method can avoid a doctor’s second opinion on the lesion and reduce the diagnosis time.

### 5.3. Loss Validity

We compared our proposed hybrid loss with several common loss functions in segmentation tasks and quantified these four kinds of losses in Table 6. The results show that the performance of the hybrid loss function is competitive because the hybrid loss, which combines binary cross entropy (BCE) loss and dice coefficient loss (DL), is suitable for brain MRI image segmentation tasks with imbalanced positive and negative samples.

### 5.4. Analyze the Robustness of the Proposed Method to Noise

**Figure 7:**
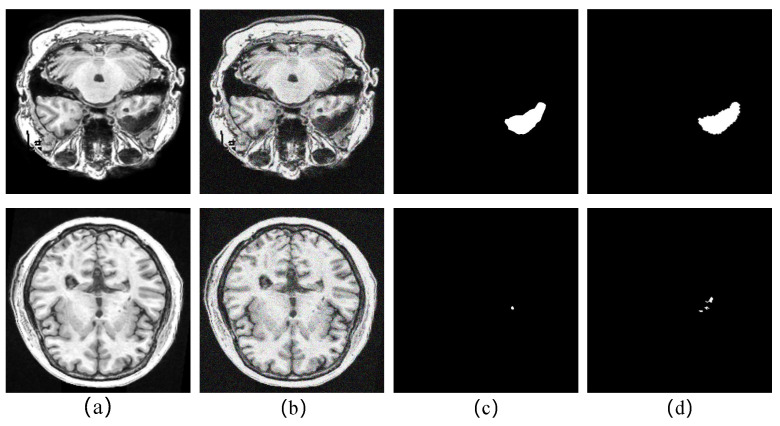
Qualitative analysis of the degradation of the model’s performance after adding noise: (**a**) original image; (**b**) Gaussian noise-processed image; (**c**) segmentation result of noisy image; (**d**) ground truth.

The noise in real-world biomedical images is a well-known problem that reduces the accuracy of diagnostics. In order to verify the robustness of our proposed model to noise, we processed images of large, medium, and small tasks with Gaussian noise, and we set the σ of Gaussian noise to 40 to make the noised image more realistic. Table 7 shows the performance changes of the model in the main indicator DSC before and after adding noise. We can observe that the segmentation performance of each task drops after noise addition, especially the small object task, which drops by 23.25%. This is because, after noise enhancement, the context information of each pixel is mixed with noise information, leading to deviations in the high-level semantic information encoded by the model. Context information plays an important role in small target localization tasks. As shown in Figure 7, the model is prone to the problem of missing localization in noisy small target tasks, but it is undeniable that FECC-Net still shows excellent performance in the task of dealing with noisy large objects; this is because the squeeze-and-excitation blocks in the encoder play a role in suppressing the noise.

### 5.5. Analyze the Segmentation Process of the Proposed Method

**Figure 8:**
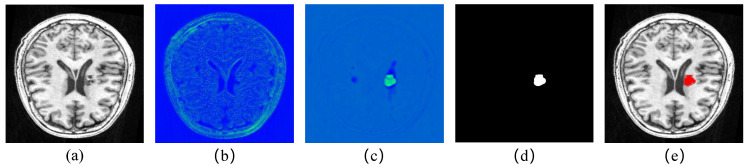
Segmentation processed attained with FECC-Net for a sample test image: (**a**) original image; (**b**) output of the first encoder block; (**c**) convolution output of the last decoder block; (**d**) segmented result; (**e**) segmentation result mapped back to the original image.

Figure 8 shows the process of FECC-Net segmentation, where Figure 8b is the output of the first encoder block. From the figure, it can be observed that the ability of our designed encoder block to extract shallow apparent features is outstanding. The figure even depicts boundary features not visible to the naked eye. Figure 8c is the convolutional output of the last decoder block; we can observe that this feature map concentrates more weight on the lesion location, proving the gain effect of hybrid loss during the backpropagation of the network. Figure 8d is the segmentation result; we hoped to locate the lesion area in the segmentation task, so we set the lesion as the foreground (binary 1) and other parts as the background (binary 0) by a sigmoid function [42,43]. Finally, the binary image was evaluated and confirmed according to the GT image. To be more in line with the visualization effect of practical applications, we mapped the segmentation results back to the original image for comparison, as shown in Figure 8e.

## 6. Discussion

In this section, based on the experimental results above, we discuss the effectiveness and researchability of segmentation in brain MRI images through deep learning models:

Effectiveness of deep learning models for segmentation of brain MRI images. In the Introduction section of this paper, we listed and analyzed the current brain MRI segmentation methods, which are mainly divided into threshold methods based on image processing and methods based on deep learning. It is well known that threshold methods cannot effectively segment the results under conditions of large amounts of noise. Therefore, more and more scholars have recently adopted U-Net for segmentation in brain MRI images and proved the effectiveness of deep learning models in brain MRI images. However, the current deep learning model relies too much on the ontology structure of U-Net, and there are problems, such as insufficient context information extraction and large model parameters. Our research proposes a light encoder–decoder framework and feature enhancement module, which reduces the parameters while improving the model’s accuracy, proving that the medical image segmentation model can achieve the requirements of high precision and small parameters. Such a baseline is more realistic for application to life.

Researchability of deep learning models in brain MRI segmentation. In the Experimental section, we conducted a comparison experiment on the test samples before and after adding noise. It can be observed that the noise still interferes with the deep learning model, especially for targets that are difficult to focus on with the naked eye. Therefore, some works combine semantic segmentation and image denoising to achieve high-precision semantic information classification [44]. We plan to apply related ideas to brain MRI segmentation in future work. In the analysis of the model segmentation process in Section 5.5, the research content of this paper is the precise localization of lesions. Our next effort will be to analyze the uncertainty based on the location of brain MRI lesions, which will help doctors to judge the prediction results. In recent years, uncertainty analysis methods have emerged in the field of medical imaging [45,46,47], but how to implement an uncertainty analysis in lightweight models still needs further exploration, and we are also conducting research related to this.

## 7. Conclusions

This paper proposes a novel feature enhancement and context capture network (FECC-Net) based on brain MRI images. We achieved low-density cerebral infarct lesion localization on the ATLAS dataset and compared FECC-Net with current medical image segmentation models. FECC-Net reached the leading position for all indicators and, most especially, is 8.97% higher than DoubleU-Net (the second-ranked method) in DSC and 9.78% higher in mIoU. FECC-Net is currently the medical image segmentation model with the smallest total network parameters, only 7.0M, which is half of U-Net. It is worth mentioning that our proposed FECC-Net-lite achieves as high as 0.6353 on DSC, which leads DoubleU-Net by 4.61%. The experimental results of FECC-Net-lite show that: (1) The lightweight encoder–decoder architecture can be used as a baseline for medical image segmentation, and (2) feature enhancement plays an indispensable role in the performance optimization of medical image segmentation. We believe that these encouraging results from FECC-Net will become the baseline for brain MRI image segmentation.

## Figures and Tables

**Table 1:** The quantitative comparison of different methods. FECC-Net-lite indicates unused enhanced encoder.

Method	DSC	Recall	Precision	Total Parameters
SegNet [32]	0.2767	0.2532	0.3938	29.5 M
PSPNet [33]	0.3571	0.3335	0.4769	48.1 M
U-Net [8]	0.4606	0.4449	0.5994	15.1 M
Deeplab v3+ [34]	0.4609	0.4491	0.5831	41.3 M
ResUNet [35]	0.4702	0.4537	0.5941	33.2 M
2D Dense-UNet [36]	0.4741	0.4875	0.5613	50.0 M
X-Net [37]	0.4867	0.4752	0.6000	15.1 M
TransUNet [14]	0.5855	0.5796	0.6352	32.3 M
DoubleU-Net [13]	0.5892	0.5878	0.6345	29.2 M
FECC-Net (ours)	**0.6789**	**0.6856**	**0.6978**	7.0 M
FECC-Net-lite (ours)	0.6353	0.6372	0.6677	**3.8 M**

**Table 2:** Quantitatively compare the proposed method to the current best results on small targets.

Method	DSC	mIoU	Recall	Precision
ResUNet [35]	0.3464	0.2467	0.2807	0.3051
X-Net [37]	0.4358	0.3581	0.3960	0.4297
TransUNet [14]	0.4590	0.3151	0.5188	0.5328
DoubleU-Net [13]	0.4750	0.3212	0.5246	0.5691
FECC-Net (ours)	**0.5159**	**0.3657**	**0.5608**	**0.6010**

**Table 3:** Quantitative comparison of the proposed method to the current best results on medium targets.

Method	DSC	mIoU	Recall	Precision
ResUNet [35]	0.6185	0.4563	0.5960	0.6273
X-Net [37]	0.6696	0.5127	0.6924	0.7035
TransUNet [14]	0.6899	0.5567	0.7159	0.7748
DoubleU-Net [13]	0.6890	0.5580	0.7103	0.7611
FECC-Net (ours)	**0.7195**	**0.5969**	**0.7701**	**0.7777**

**Table 4:** Quantitative comparison of the proposed method to the current best results on large targets.

Method	DSC	mIoU	Recall	Precision
ResUNet [35]	0.7823	0.6464	0.7401	0.8397
X-Net [37]	0.8131	0.7707	0.8127	0.8677
TransUNet [14]	0.8834	0.8084	0.8987	0.8917
DoubleU-Net [13]	0.8887	0.8172	0.8835	0.9159
FECC-Net (ours)	**0.9072**	**0.8311**	**0.9034**	**0.9246**

**Table 5:** Quantitative analysis of the four enhanced encoders.

Enhanced Encoder	DSC	mIoU	Recall	Precision
VGG19 [38]	0.6620	0.5836	0.6674	0.6958
ResNet [39]	0.6627	0.5898	0.6640	0.6942
DenseNet [40]	0.6632	0.5782	0.6701	0.6791
Ours	**0.6789**	**0.6107**	**0.6856**	**0.6978**

**Table 6:** Quantitative analysis of the four loss functions.

Method	DSC	Recall	Precision
Focal Loss [41]	0.6460	0.7818	0.5772
Binary Cross Entropy Loss	0.6412	0.6782	0.6391
Dice Coefficient Loss	0.6355	0.6751	0.6302
Hybrid Loss (ours)	**0.6789**	**0.6856**	**0.6978**

**Table 7:** Comparison of the main indicators’ DSC before and after adding noise.

Indicator	Small Target Task	Medium Target Task	Large Target Task
DSC (before adding noise)	0.5159	0.7195	0.9072
DSC (after adding noise)	0.3188	0.6176	0.8607

## Data Availability

The ATLAS(MRI data) that support the findings of this study are openly available in INDI-Retrospective at http://fcon_1000.projects.nitrc.org/ (accessed on 24 May 2022).

## Abbreviations

The following abbreviations are used in this manuscript:

FECC-Net	Feature Enhancement and Context Capture Network
ASPP	Atrous Spatial Pyramid Pooling
ATLAS	Anatomical Tracings of Lesions After Stroke
MRI	Magnetic Resonance Imaging
CNNs	Convolutional Neural Networks
FCN	Fully Convolutional Network
BCE	Binary Cross Entropy
HL	Hybrid Loss
